# Guidance of Signaling Activations by Cadherins and Integrins in Epithelial Ovarian Cancer Cells

**DOI:** 10.3390/ijms17091387

**Published:** 2016-08-23

**Authors:** Francesca Roggiani, Delia Mezzanzanica, Katia Rea, Antonella Tomassetti

**Affiliations:** Unit of Molecular Therapies, Department of Experimental Oncology and Molecular Medicine, Fondazione IRCCS Istituto Nazionale dei Tumori, Via Amadeo 42, Milan 20133, Italy; francesca.roggiani@istitutotumori.mi.it (F.R.); delia.mezzanzanica@istitutotumori.mi.it (D.M.)

**Keywords:** epithelial ovarian cancer, adhesion, cadherin, integrin, signal transduction, proliferation, migration, invasion

## Abstract

Epithelial ovarian cancer (EOC) is the deadliest tumor among gynecological cancer in the industrialized countries. The EOC incidence and mortality have remained unchanged over the last 30 years, despite the progress in diagnosis and treatment. In order to develop novel and more effective therapeutic approaches, the molecular mechanisms involved in EOC progression have been thoroughly investigated in the last few decades. At the late stage, peritoneal metastases originate from the attachment of small clusters of cancer cells that shed from the primary site and carried by the ascites adhere to the abdominal peritoneum or omentum. This behavior suggests that cell–cell or cell–matrix adhesion mechanisms regulate EOC growth and dissemination. Complex downstream signalings, which might be influenced by functional cross-talk between adhesion molecules and co-expressed and activated signaling proteins, can affect the proliferation/survival and the migration/invasion of EOC cells. This review aimed to define the impact of the mechanisms of cell–cell, through cadherins, and cell–extracellular matrix adhesion, through integrins, on the signaling cascades induced by membrane receptors and cytoplasmic proteins known to have a role in the proliferation, migration and invasion of EOC cells. Finally, some novel approaches using peptidomimetic ligands to cadherin and integrins are summarized.

## 1. Introduction

Epithelial ovarian cancer (EOC) is a devastating disease with an overall five-year survival rate of approximately 45% [1,2]. EOCs are usually diagnosed when malignant cells have already invaded the peritoneal cavity and, although most of the patients are sensitive to the first line chemotherapy, 50% of them relapse with a chemoresistant disease. For all these reasons, EOCs are the fifth main cause of cancer-related deaths among women, and the primary cause of death from gynecological cancer [3]. Therefore, in cancer research, investigations aiming to clarify the mechanisms of EOC tumorigenesis and progression are one of the most important areas. EOC group different diseases with a common anatomical location [4] but display high molecular and etiological heterogeneity [5,6,7,8].

EOCs are divided into two large groups [9] designated types I, genetically stable and not very aggressive [10], and type II, genetically unstable and very aggressive tumors, which are usually diagnosed at the advanced-stage. Type II tumors include high-grade serous, high-grade endometrioid, malignant mixed mesodermal tumors (carcinosarcomas), and undifferentiated carcinomas being the serous high-grade ovarian carcinoma (HGSOC) the most representative tumors [11].

More than 50% of EOC patients at the late stage, in particular those with type-II tumors, present with ascites/effusions in their abdominal cavity rich of tumor cells [12]. The ascites are accumulated since implanted tumor cells give rise to the obstruction of lymphatic vessels, preventing the outflow of fluid that transpires from the tumor vessels. Hence, patients affected by HGSOC type-II tumors have the peritoneal cavity invaded by metastatic tumors, growing in the solid stromal matrices, and multicellular aggregates (MCAs) floating and growing in the malignant ascites [13]. These MCAs overcome anoikis [14] and persist as ascites [15]. Although a possible mechanism of hematogenous HGSOC metastasis formation to the omentum has also been reported [16], the general consensus is that these MCAs originate by the shedding of malignant cells into the peritoneum from the primary tumor and that disaggregation and attachment to the sub-mesothelial extracellular matrix (ECM) allow the formation of secondary lesions [17,18,19]. Once adhered to the peritoneum, EOC cells proliferate, migrate and invade the surrounding tissues. An elegant approach of a live image-based in vitro model determined that a myosin–generated force allows EOC MCAs to displace and remove the mesothelial monolayer. This process is now known as mesothelial cell clearance [20]. The EOC metastasis outgrowth occurs upon the remodeling of cell–cell adhesion molecules (i.e., cadherins) during spheroids dis-aggregation. In addition, the integrins expressed on the surface of EOC cells are essential to the attachment of EOC cells to the sub-mesothelial ECM [18,19,20,21].

Interestingly, a proteomic approach for MCA analysis for EOC patient stratification has been able to identify three adhesion-related subsets with potential predictive impact [22]. These data highlight the influence of the adhesion molecules in the clinical EOC behavior.

Overall, the processes of EOC progression require that cell–cell, through cadherins, and cell–ECM adhesion, through integrins, cooperate, directly or indirectly, to the activation of signaling pathways relevant to the proliferation/survival and the migration/invasion mechanisms of EOC cells.

## 2. Cadherin-Associated Signaling Activation

**Figure 1:**
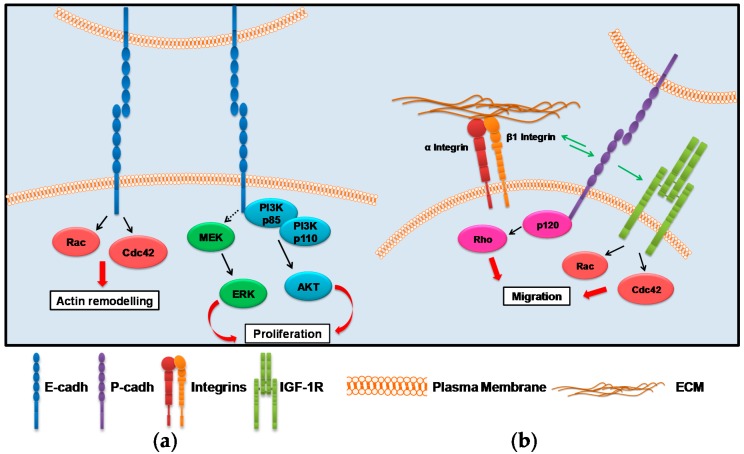
Schematic representation of (**a**) E- and (**b**) P-cadh-associated signaling cascades activated in EOCs (Epithelial Ovarian Cancers). The arrows indicate: green, protein-protein interaction, black, signaling cascades; discontinuous, possible signaling cascade; red, cellular effects. The abbreviation used are: cadh, cadherin; IGF-1R, Insulin Growth Factor Receptor 1; ERK, Extracellular signal-Regulated Kinase; MEK, MAP or ERK kinase; PI3K, phosphoinositide 3-kinase; p120, p120 catenin. Rho, Rac and CDC42 are Rho-GTPases.

Cell–cell adhesion is mediated by cadherins (cadhs) through the calcium-dependent homophilic interaction of their extracellular domain to form, associated with cytoplasmic proteins (β- and α-catenin (ctn) together with p120ctn) intercellular structures called adherens junctions (AJs) (see, for review, [23]). The AJs constitute a physical bridge between the cadherin complex and the cortical actin filaments thus regulating cellular dynamic behaviors, such as rearrangements, movement and shape changes during embryonic development as well as during neoplastic transformation and progression. The cytoplasmic tail of cadhs has no catalytic activity; therefore, possible signaling activation must occur upon the recruitment of signaling molecules to the site of the cadh–ctn complex [24]. In epithelial tissues, the homophilic E-cadh ligation can activate the junctional GTPase signaling downstream to Rac [25,26,27,28] and Cdc42 [26,29]. E-cadh can therefore regulate the localization and function of the Rho GTPase with a mechanism described as ‘outside-in signaling’ [30,31] (Figure 1a).

Below, E- and P-cadh-associated signalings are discussed. N-cadh displays similar structural and functional properties to E-cadh (see, for review, [32]). In EOCs, N-cadh expression is associated with a more aggressive and chemoresistant phenotype [33], but its role in modulating signaling activation is still unclear.

### 2.1. E-cadh

In cancer, the switch in expression of E-cadh to N-cadh or the up-regulation of cadh-11 and P-cadh are important indicators of progression and are believed to facilitate the epithelial–mesenchymal transition (EMT), which leads to a more migratory and invasive phenotype [34,35]. In metastatic solid tumors, E-cadh expression is usually lost in human breast, bladder, lung and pancreatic carcinomas, which nevertheless express other cadherins, especially N-cadh [36]. For this reason, E-cadh has been proposed as a tumor suppressor since, in invasive carcinoma cells, ectopically expressed E-cadh was associated with a decreased growth potential [37]. In confluent cells, E-cadh was shown to be associated to epidermal growth factor receptor (EGFR) leading to receptor immobilization and alteration of the ligand-receptor affinity, thus inhibiting the signaling activation [38,39].

Very recent publications are beginning to revisit the general consensus that EMT is necessary for the metastasis formation occurring when the tumors become more malignant. In these reports, E-cadh expression was found maintained during the formation of lung metastasis in mouse models of breast and pancreatic cancers [40,41]. These data are not surprising since E-cadh lost was previously showed to be a rare event in both invasive ductal [42] and inflammatory breast carcinomas [43]. Mechanistically, in a breast carcinoma patient-derived 3D organoid model, E-cadh homophilic adhesion was present in a leading cell population and necessary during invasion of collagen I gel [44,45].

In EOCs, initial immunohistochemical analysis revealed low E-cadh expression in advanced-stage disease [46,47]. Data made on a higher number of EOC biopsies assessed that E-cadh is still expressed at cell–cell contacts in late stage tumors [48,49,50,51]. Furthermore, in EOC MCAs, the predominant cadherin expressed at cell–cell contact is E-cadh (our unpublished results and [52]) and contributes to aggregate formation [53]. E-cadh was first assessed to upregulate ligand-independent EGFR trans-phosphorylation leading to AKT and MAPK activations [54] (Figure 1a). Furthermore, E-cadh-mediated AJ formation recruits PI3K-p85 to the cell membrane contributing to activation of the PI3K/AKT pathway and to the up-modulation of EOC growth [55]. In the EOC cell line SKOV3, E-cadh functionality can also increase mitogen-activated protein kinase/extracellular-signal regulated kinase (MEK/ERK) activation, although no physical association with MEK or even EGFR was demonstrated [56] (Figure 1b).

While in normal epithelial cells E-cadh is maintained on the membrane by p120 catenin and its levels are controlled through the endocytic pathway [57], in transformed cells, E-cadh loss is also due to the proteolitic cleavage of the extra-cellular domain resulting in a 80-KDa fragment. This soluble (s) E-cadh might be generated though the action of metalloproteases (MMTs), such as ADAM10 and MMPs [58,59] usually upregulated in cancers, or γ-secretases, like presenilin 1 [60]. The 80 KDa soluble form of E-cadh has been found in the serum of cancer patients with poor prognosis [61]. In EOCs, the levels of sE-cadh in cystic fluid from patients presenting with complex ovarian masses was higher than in patients presenting with benign tumors [60]. sE-cadh was found in the EOC ascites, and in vitro experiments demonstrated that the integrin clustering occurring during the adhesion to the sub-mesothelial collagen I stimulated EGFR-dependent MMP9 expression [62], thus inducing sE-cadh production [63]. These data suggest that EGFR activation and production of sE-cadh constitute a feedback loop able to promote proliferation and/or migration/invasion. More recently, the 85-KDa and 23-KDa E-cadh, produced by calpain, were found in solid metastatic peritoneal masses of advanced EOC patients [64]. The presence of the 85-KDa E-cadh fragment correlated with a worse patient outcome, and the authors hypothesized that the 85-KDa fragment might enable tumor cluster formation and peritoneal dissemination [64].

### 2.2. P-cadh

Among cadherins, P-cadh has a proven role during the progression of several carcinomas, as cervix [65], esophagous [66], breast [67] and colon [68], including EOCs [69]. In EOC, an increase in P-cadh expression in the tumor masses, concomitant with a decrease of E-cadh expression, is associated with a progression from stage I to stage II tumors [69]. The pro-metastatic role of P-cadh is mainly due to a cross-talk with the axis constituted by the cytoplasmic p120ctn and the activation of the Rho GTPase signaling. In this context, once Gonadotropin-releasing hormone (GnRH), whose physiologic role is the control of pituitary gonadotropin secretion, induces the E- to P-cadh switching, p120ctn moves from the membrane to the cytoplasm, thus leading to activation of mechanisms of migration and invasion [70]. The role of p120ctn as determinant of migration upon GnRH stimulation is due to a trans-activation of insulin-like growth factor-1 receptor (IGF-1R) by P-cadh, which induces Rac1 and Cdc42 activations [71].

P-cadh also contributes to the adhesion of EOC cells to the peritoneum and in the metastasis formation and has been considered the predominant cadherin subtype expressed in the MCAs of the peritoneal effusion [69]. Furthermore, its inhibition reduced tumor growth, ascites formation and metastatis in in vivo pre-clinical models [72]. Besides its role in the MCA formation, P-cadh can also contribute to inhibition of anoikis. Accordingly, in vitro P-cadh knockdown increases cell death of EOC cells growth in suspension and in vivo decreases the dissemination of EOC cells injected in the peritoneum of immunocompromised mice [73], once again through a cross-talk with p120ctn and Rho GTPase activation. Another report has recently showed a P-cadh role in the MCA formation through a cross-talk with β1 integrin. In particular, P-cadh induces the Golgi glycosyltransferase (ST6Gal-1), which mediate β1 integrin hypersialylation, a post-translational modification that results in maturation of this integrin and activation of the downstream signaling [74] (Figure 1b).

## 3. Integrin-Associated Signaling Activation

Integrins are αβ heterodimeric transmembrane proteins implicated in numerous physiological processes including adhesion to the extracellular matrix, proliferation, survival, migration and differentiation [75]. The molecular and physical interactions among the cells and with ECM can strongly affect cell behavior, thus inducing cancer cell invasion and dissemination. A group of integrins recognizes and binds to the Arg-Gly-Asp (RGD) motif present on the ECM proteins, and the specificity of integrin binding to different ECM proteins is determined, in part, by other amino acids surrounding the RGD sequence [76]. As already said for the cadherins, ntegrin cytoplasmic tails do not exert a kinase activity but instead are able to activate specific intracellular kinases, such as FAK (Focal Adhesion Kinase), which, in turn, recruit the src kinase [77]. Src phosphorylates a number of FAK-associated proteins including paxilin, tensin and the adaptor p130CAS (Crk-Associated Substrate). FAK activation also leads to the recruitment of other SH2-containing proteins, including the PI3K, PLC-γ and the adapter proteins Grb7 [77] and Grb2 to partially mediate ERK activation [78]. Finally, the FAK/Src complex modulates the activity of small GTPases leading to the actin cytoskeleton remodeling necessary for cell adhesion and migration [79].

In EOC, the expression of ECM components and integrins has been found to be involved not only in the cell detachment from the primary tumor and from the peritoneal metastasis [80,81,82], but also in the resistance to anoikis of the EOC spheroids [83,84]. Dysregulation and aberrant deposition of ECM components, due to the activation of different MMPs [85], contribute to tumor progression. The expression of vitronectin [80] and fibronectin [86] at the periphery of mesothelial cells actively participates in EOC cell motility and metastasis. In addition, fibronectin has been also shown to mediate EOC cell migration and invasion through the upregulation of the FAK/PI3K/Akt pathway [87] and is an indicator of poor prognosis in invasive EOC [88].

### 3.1. Integrins and Receptor Tyrosine Kinase (RTKs)

**Figure 2:**
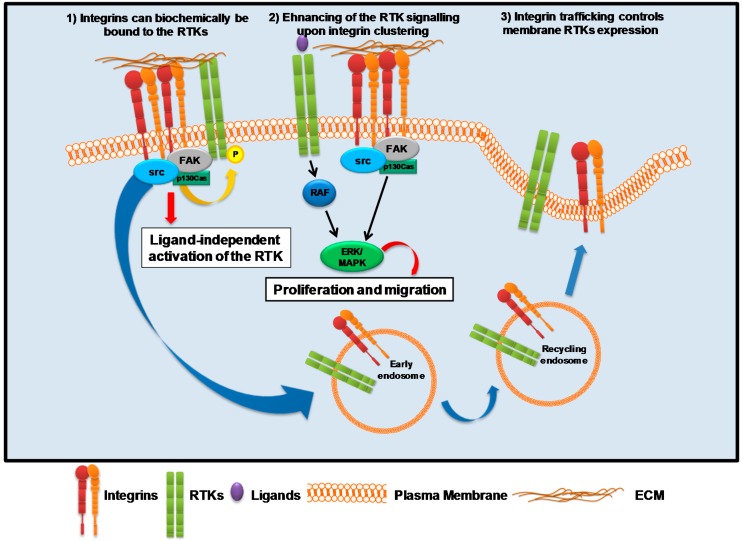
Schematic representation of the the signaling cascades activated in cancer cells by the cross-talk between integrins and RTKs. The arrays indicate: black, signaling cascades; yellow, phosphorylation; discontinuous, possible signaling cascades; blue, trafficking; red, effect. The abbreviations are: RTK, Receptor Tyrosine Kinase; ECM, Extra Cellular Matrix, MAPK, Mitogen Activated Protein Kinase, FAK, Focal Adhesion Kinase; src, Sarcoma viral oncogene; ERK, Extracellular signal-Regulated Kinase.

Numerous studies suggest that the cooperation between integrins and RTKs exists and exerts a central role in cancer progression regulating invasion, proliferation and survival (see, for review, [89]). Considering that neither α or β subunit possess catalytic activity, it is possible that multiple mechanisms may regulate the cross-talk between integrins and RTKs. Three main types of integrins/RTKs interaction have been identified [90] (Figure 2): (1) integrins can physically bind to RTKs; (2) integrins clustering upon the binding to ECM can enhance signaling pathways that are activated following ligand-dependent RTKs activation; and (3) integrins and RTKs reciprocally control their surface expression.

The RTK represents the largest family of oncogenes, and the inhibitors targeting these receptors have already shown clinical efficacy as anti-cancer agents in solid tumors other than EOCs [91]. RTKs have been found to be over-expressed in EOCs as compared to normal counterparts [92].

#### 3.1.1. EGFR

The EGFR is a transmembrane glycoprotein receptor that belongs to the ErbB family of RTKs. The ligands for the EGFR are the EGF, the betacellulin (BTC), TGF-α, amphiregulin (AR), and epiregulin (EPR) [93]. EGF ligands can induce the homodimerization of EGFR as well as the heterodimerization with other members of the family: HER2, HER3 and HER4 [93].

In solid tumors, EGFR can interact with many integrins such as β1 [94], α6β4 [95] and αvβ3 [96,97], likely by forming a multimeric complex that also includes Src and the adaptor protein p130Cas [96]. This complex is necessary for the ligand-independent activation of the EGFR leading to signaling involved in cell survival and proliferation in response to ECM [94]. EGFR is expressed in up to 70% of EOCs [98], and its activation contributes to cell proliferation, invasion, angiogenesis and resistance to apoptosis [99]. EGFR activation can also induce a pro-inflammatory program with the co-expression of IL-6 and PAI-1 in a subset of EGFR-expressing EOCs with shorter progression-free survival after chemotherapy [100]. A direct correlation between EGFR and integrin expression has been reported. Indeed, reduced expression of EGFR in NIH:OVCAR-8 cells was associated with reduced integrin α6, a laminin-1 receptor, and cell adhesion with the down-modulation of MMP-9 activity, thus reducing the aggressiveness of EOC cells [101]. Conversely, the expression of a constitutively active EGFR has been proposed to contribute to a more aggressive disease throughout the down-modulation of the integrin α2 expression, which leads to changes in cell shape and focal adhesion formation [102].

#### 3.1.2. c-MET

c-MET oncogene is a membrane receptor that is essential for embryonic development and wound healing [103]. The natural ligand for this receptor is the hepatocyte growth factor, HGF, whose binding to the c-MET receptor leads to receptor phosphorylation and activation [103].

As in other carcinomas, also in EOCs, Sawada et al. found that the over-expression of c-MET was associated with the worst prognosis [104]. The knockdown of c-Met expression by small interfering RNA (siRNA) induced a decreased activation of the MEK/ERK and PI3K/AKT signaling pathways and reduced adhesion, invasion, peritoneal dissemination, and tumor growth through the inhibition of the expression of α5β1 integrin [104]. Furthermore, α5β1 integrin binding to the fibronectin also leads to a ligand-independent activation of c-MET signaling to FAK and Src. This mechanism is due to a biochemical association between α5 integrin and c-MET [105]. Since it has been reported that cancer-associated mesothelial cells that colonize the peritoneum produce fibronectin [86], able to trap HGF produced by the fibroblasts of the tumor microenvironment [106], the inhibition of c-MET activation seems to be a promising target to block cancer metastasis. Recently, Moran-Jones et al. investigated the effect of a novel ATP competitive inhibitor, INC280 (Novartis, Basel, Switzerland), which selectively inhibits c-MET activation in a number of EOC in vitro models [107], also decreasing the migration of EOC cells adherent to ex vivo peritoneal tissue.

#### 3.1.3. VEGFR Family

For EOC patients, targeting angiogenesis is a novel therapeutic option, which includes the use of bevacizumab, an antibody that binds to VEGF, thus inhibiting its association with the receptor [108]. The VEGF and the VEGFR are expressed by EOC cells, and increased VEGF expression has been associated with the tumor progression and poor survival [109,110].

The interaction between the VEGFR and integrins is very important for angiogenesis. In particular, ligand-dependent VEGFR-2 phosphorylation activates c-src which then phosphorylates the cytosolic tail of β3 integrin, promoting a feedback loop with the formation of the VEGFR-2/ανβ3 complex and increased integrin and VEGFR-2 signaling [111].

In in vivo EOC models, the combination of anti-angiogenic and anti-integrin combination therapy using Bevacizumab and etaracizumab (an antibody that binds to the integrin αvβ3 inhibiting its activation) was more effective in inhibiting tumor growth and microvessel density than individual drugs [112]. In particular, the etaracizumab was reported to be particularly efficient in reducing the volumes of VEGFR-dependent SKOV3ip tumors in nude mice [113]. These findings might open the possibility that a dual blockade of VEGF and the αVβ3 integrin in combination with chemotherapy may impair EOC growth in those patients whose tumors display high vessel density.

#### 3.1.4. Axl

Deregulation of the receptor tyrosine kinase Axl, a member of the TAM RTK family, which also includes Mer and Tyro3, has been implicated in aggressive phenotype, tumor metastases and the progression of several human cancers [114]. The ligand of TAM RTKs, Gas6, displays the highest activity for Axl, which has been proposed as a therapeutic target since its genetic and pharmacologic inhibition could prevent or overcome acquired resistance to EGFR inhibitors [115]. Indeed, an EMT signature including Axl was identified and associated with the resistance to EGFR and PI3K inhibitors [116]. Multiple downstream signaling pathways, including PI3K/Akt and MAPK, can be elicited by Axl/Gas6 in different cell types [117,118].

Axl expression increased in type II EOC primary tumors and metastases compared to the ovarian surface epithelium [119] and Gas6 has been identified as prognostic marker in HGEOCs [120]. Therapeutic blockade of Axl activation reduced the migratory and invasive capability of HGSOC cells in vitro and in xenograft models [121]. Recently, the signaling elicited by Axl activation was dissected showing a cross-talk with the integrin/ECM pathway through the adaptor protein p130Cas leading to Rac activation [122]. Furthermore, an Axl-driven 61-gene signature, which included several collagens and ECM-associated proteins, was associated to the most aggressive HGSOC subtype [122].

### 3.2. Integrins and RNAseT2

A novel factor that may play a role in the complex integrin signaling is the protein RNASET2, a ribonuclease that belongs to the T2 family. This protein, known to be downregulated in EOC and EOC cell lines [123], has been found to display tumorigenic and metastatic suppressor properties, independent from its ribonuclease activity, in vivo [123,124,125,126]. Recently, a new role of this protein was discovered that led to the hypothesis than RNASET2 may affect the integrin signaling pathway. RNASET2 proved to have the capability to reorganize the actin cytoskeleton and consequently influence cells motility, invasion capability and cell adhesion to ECM. In fact, silencing of the protein in the EOC cell line NIH-OVCAR3 triggered a disruption of the network of actin filaments and stress fibers, inducing a pattern of peripheral actin filaments [126], thus leading to increased migration and adhesion to laminin, collagen I and IV. Of note, the adhesion of these cells on collagen I was accompanied to an increase of paxillin activation and, accordingly, to an increase of mature focal adhesions (FAs) [126]. Although RNASET2 and tr-T2-50, a recombinant truncated form of the human RNASET2 deprived of its RNAse activity [127,128], showed the capability to bind actin in vitro, Acquati et al. did not find any co-localization between the actin cytoskeleton and RNASET2 in NIH-OVCAR3 cells [126], supporting the hypothesis of a cross-talk with the integrin activation and consequent re-organization of the actin cytoskeleton. Conversely, RNASET2 resulted in being significantly down-regulated in drug-resistant EOC biopsies and EOC cell lines, suggesting an involvement of this protein in the response to chemotherapy [129]. Since integrin signaling pathway is also associated with therapy resistance in cancer (see, for reviews, [130,131]) it can be assumed that RNASET2 may act on integrin signaling pathway, not only influencing FA dynamics, cell motility and adhesion on ECM, but also influencing downstream PI3K/AKT pathwaya and, in turn, drug resistance.

## 4. Targeting Cell Adhesion Using Peptidomimetic Ligands

Besides the use of specific antibodies, in the last few years, the possibility of designing small molecules acting as antagonists or agonists of the interactions between cell/ECM or cell/cell and the consequent intracellular signalings has been developed. Peptidomimetic integrin ligands have proved to be valuable for tumor directed delivery of diagnostics or therapeutics [132,133,134,135]. A subset of integrins (8 out of 24) recognizes RGD sequence and several RGD peptide mimetics entered in clinical trials as integrin-targeted agents in EOC [136]. Integrin ligands tested in tumor cells are summarized in Table 1. The cilengitide, designed against the αvβ3, has been considered the most promising compound; however, the phase III clinical trials failed likely due to a low specificity, metabolic instability and low biodistribution [137,138]. To accomplish an adequate biodistribution and uptake in cancer cells, new compounds were obtained by the coniugation between the peptidomemitic ligands and a drug or a nanoparticle made of new materials (see, for review, [139]).

As far as the cadherins are concerned, some short peptide sequences, corresponding to the binding domain of N-cadh, have been shown to disrupt cell adhesion-induced apoptosis in an antagonist manner [140] (Table 1). In cancer cells in vitro and in xenotransplanted animal models, one of these small peptides, the ADH-1, enhanced antitumor activity of melphalan-treated melanomas [141] by altering both homotypic (between cancer cells) and heterotypic (between cancer cells and surrounding endothelial cells) cadherin interactions. Although ADH-1 has been well tolerated in phase I and II clinical trials, its antitumor efficacy as a single agent has been moderate [142,143]. This is probably due to its low affinity for N-cadh and the lack of stability in biological fluids. Very recently, Turley et al. assessed that ADH-1 may have a dichotomous effect: it can increase tumor growth rate and sensitivity to some chemotherapy agents by increasing AKT activation and, on the other side, ADH-1 facilitates drug delivery, enhancing the vascular permeability [144]. More recently, some peptidomimetics able to bind at the sites of the homophilic N-and E-cadh binding have been shown to inhibit cell–cell adhesion [145] and E-cadherin-mediated AKT phosphorylation of EOC cells [146].

## 5. Conclusions

The present review has reported the more recent knowledge on the cooperation of cell–cell and cell–ECM adhesion molecules, such as cadherins and integrins, as well as their elicited mechanisms, together with membrane or cytoplasmic signaling molecules that are required for the growth, migration and invasion of EOC cells, especially taking into account those mechanisms necessary for EOC intra-peritoneal dissemination. The reported information suggests that different cadherins and integrins could affect several aspects of EOC progression. For example, E-cadh is important in spheroid formation, but, at the late stage, the switch in cadherins expression could enhance metastasis through a different pattern of integrins at the plasma membrane.

Recent evidence shows that mono-targeting, particularly of RTKs, inefficiently impacts tumor growth control, suggesting that a multi-target treatment might be more efficient. As we have shown here, RTKs might require specific integrins for full activation, and for their contribution to tumorigenesis and metastasis formation, it may be plausible to tailor the use of integrin antagonists in combination with RTK inhibitors. For instance, inhibitors to adhesion molecules together with target-specific drugs could be effective as adjuvant therapy in advanced-stage EOC patients. Peptidomimetics to E-cadh could be relevant for EOC relapsing patients presenting E-cadh-expressing MCAs, and labelled E-cadh binding compound/s could be exploited for the detection of minimal residual disease after first debulking or during chemotherapeutic treatment. Overall, these new approaches, aimed to inhibit adhesion-dependent mechanisms, represent a challenge in the field of cancer research likely to counteract the processes of mesothelial cell clearance and MCA formation occurring during the intra-peritoneal dissemination of EOC cells. Investigations through large scale/high-throughput technologies could help to identify further relevant cross-talks that involve adhesion molecules.

Further efforts are necessary for a better understanding of the different roles exerted by the numerous players participating in these complex mechanisms for a more rational development of better treatments in the aim to improve EOC patient outcome.

## Figures and Tables

**Table 1:** Integrins or cadherins expressed on EOC cells and their suitable ligands.

Integrins	Ligands	Chemical Scaffold	Tumor Cell Model ^1^	Reference
**αvβ3**	cRGDfV ^2^	Cyclopentapeptide	GBM	[147]
	RGD4C	Cyclopentapeptide	BC	[148]
	cAbaRGD	Azabicycloalkane	EOC	[149]
	(DKP)-RGD	Dichetopiperazine	EOC	[135]
	*Ciso*DGR	CDAK 22-mer peptide	BC	[150]
	*Cyclo[DKP-isoDGR]*	Dichetopiperazine/CDAK	GBM	[151]
**α5β3**	H2009.1	20-mer peptide	NSCL-C	[152]
**Cadherins**				
**N-cadh**	N-Ac-CHAVC-NH_2_^3^	Disulphade-linked cyclic peptide	PC	[153]
**N- and E-cadh**	Compound **3**	Benzyl ring	EOC	[145]

^1^ Only ligands tested on cancer cells are reported. The abbreviations are: GBM, Glioblastoma; BC, Breast Cancer; NSCL-C Non-Small Cell Lung cancer; PC, Pancreatic cancer; ^2^ Cilengitide (Merck, Darmstadt, Germany). It also binds to α5β3 integrin; ^3^ ADH-1 or Exherin.
